# Dynamic ultrasound assessment in the diagnosis of intra-articular entrapment of the biceps tendon (hourglass biceps): A preliminary investigation

**DOI:** 10.4103/0973-6042.63212

**Published:** 2009

**Authors:** N. Pujol, R. Hargunani, S. Gadikoppula, B. Holloway, P. M. Ahrens

**Affiliations:** Department of Orthopedics, The Royal Free Hampstead NHS Trust, UK; 1 Department of Radiology, The Royal Free Hampstead NHS Trust, UK

**Keywords:** Long head of biceps, hourglass, ultrasound

## Abstract

**Background:**

The hourglass biceps, an intra-articular entrapment of the long head of the biceps (LHB), is a possible diagnosis in cases of shoulder pain associated with loss of passive elevation.

**Purpose:**

The objective of this study is to investigate the role of dynamic ultrasound (U/S) in determining the diagnosis of the hourglass biceps lesion.

**Materials and Methods:**

A prospective cohort of 16 patients with the clinical suspicion of an hourglass lesion, a preoperative ultrasound, and a confirmed hourglass LHB at surgery, were included in the study. Eight patients had preoperative dynamic ultrasound assessment of the LHB, and eight had standard ultrasound investigations and served as a control group.

**Results:**

Dynamic ultrasound accurately diagnosed an hourglass biceps in three out of eight cases. LHB hypertrophy was demonstrated in five out of eight cases with U/S and three out of eight cases with standard U/S. All patients were treated by excision of the intra-articular portion of the LHB, 15 by bipolar tenotomy, and one by LHB tenodesis.

**Conclusions:**

Dynamic ultrasound shows promise in improving the accuracy in diagnosis of LHB hypertrophy and the Hourglass lesion.

**Level of Evidence:**

III (Consecutive case-control study investigating a diagnostic test).

## INTRODUCTION

Chronic shoulder pain is often caused by pathological changes of the long head of the biceps (LHB) tendon. Inflammatory and degenerative conditions of the tendon, tears,[1] hypertrophy,[2] subluxation, and dislocation[3] have been described.

**Figure 1:**
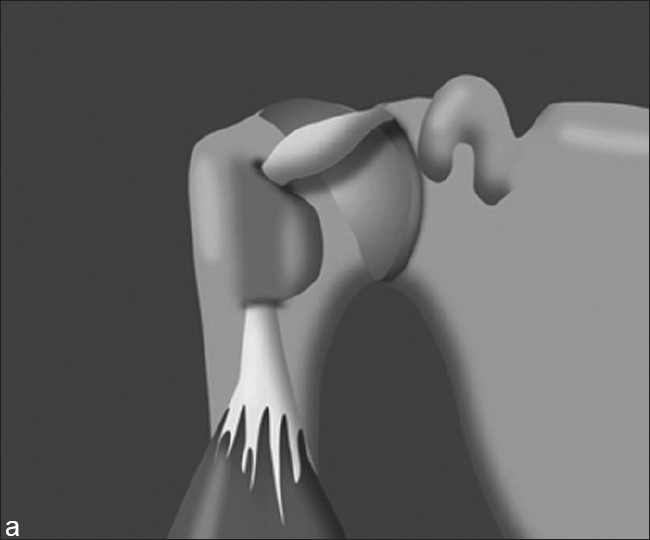
Illustration of a hypertrophic LHB with the arm at the side

**Figure 2:**
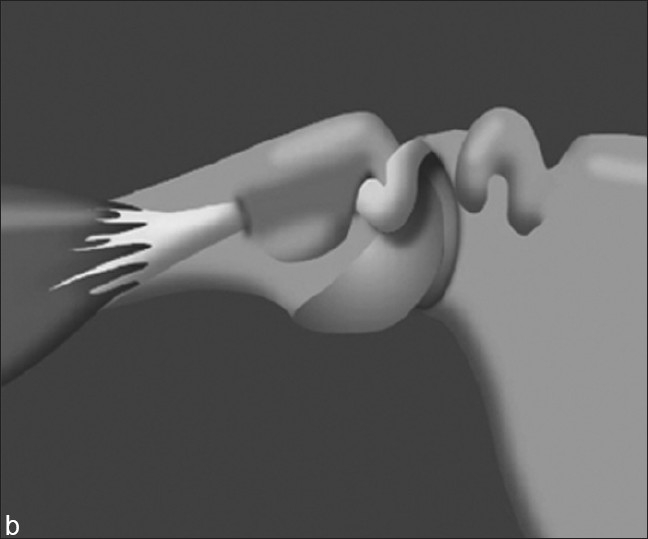
Illustration of intra-articular buckling of a hypertrophic LHB with shoulder elevation

The hourglass biceps has been described as a cause of shoulder pain and loss of motion.[4] Intra-articular entrapment of the long head of the biceps occurs in anterior elevation when the tendon cannot slide into the intertubercular groove [Figures 1 and 2]. Clinical findings are of biceps pathology with a loss of passive elevation of 10 – 20°, but with preserved gleno-humeral rotation.[4]

A variety of imaging tests have been employed in the diagnosis of lesions of the LHB. Single-contrast arthrography, double-contrast arthrography,[5] CT arthrography, ultrasound (U/S),[6–8] magnetic resonance imaging (MRI), and arthro-MRI.[9], of shoulder U/S as an imaging modality has gained widespread attention.[10,11]

Ease of comparison with the asymptomatic limb, dynamic real-time assessment, patient comfort, easy accessibility, and low cost are among the advantages of U/S. Several studies have suggested that shoulder ultrasound is useful in assessing the biceps tendon,[12–14,7] although a study of non-dynamic ultrasound evaluation concluded that it was not reliable for detecting intra-articular partial thickness tears of the LHB.[12]

Dynamic evaluation of the LHB with ultrasound was first described by Farin *et al*.,[14] suggesting that a dynamic subluxation test was reliable in the diagnosis of subluxation of the biceps tendon. This test was carried out in abduction and external rotation.

The purpose of this study was to investigate the potential of a dynamic ultrasound test in anterior elevation, for detecting the hourglass biceps lesion, in conjunction with the clinical preoperative evaluation and arthroscopic findings.

## MATERIALS AND METHODS

Over a three-year period, a prospective cohort of patients with the clinical suspicion of an hourglass biceps, preoperative ultrasound imaging, and confirmation of an hourglass biceps at surgery were studied. Sixteen patients were included (six males, 10 females). Retrospective data collection was performed and the patients included in the study were analyed. The age range was 45 to 77 years (mean 67 years). The dominant arm was affected in 13 patients.

The inclusion criteria were as follows:

- Clinical suspicion of an hourglass biceps: Anterior shoulder pain, bicipital groove tenderness, positive Speed's or Yeargason's tests, and loss of 10° to 20° of active and passive elevation.

- Imaging: Ultrasound with or without dynamic evaluation of the LHB, during passive elevation.

- Surgery: Positive hourglass test during arthroscopic procedure: Buckling and entrapment of the LHB tendon within the joint, during forward passive elevation of the arm, with the elbow extended.

### Clinical findings

All patients presented with a painful shoulder. The objective pain score evaluated by a visual analog scale averaged 6 (4 to 9). The location of pain was anterolateral in six cases, anterior in eight cases, and lateral in two cases. Palpation of the bicipital groove was tender in 12 cases. Active and passive anterior elevation averaged 120° (80 to 170°) and 150° (90 to 170°), respectively.

Eleven patients demonstrated a positive Speed's test, 12 a positive Jobe's test, and the Belly Press test was positive in three cases. impingement signs were positive in 13 cases. There was a loss of passive elevation compared with the contralateral limb in all cases.

### Ultrasonographic evaluation

The clinical diagnosis was available to the radiologist during U/S. All patients were scanned with a 7-12 MHz linear transducer on either a HDI 5000, Philips ATL (Bothell, Wash.) or a Logiq 9, GE medical Systems (Milwaukee, WI).

**Figure 3:**
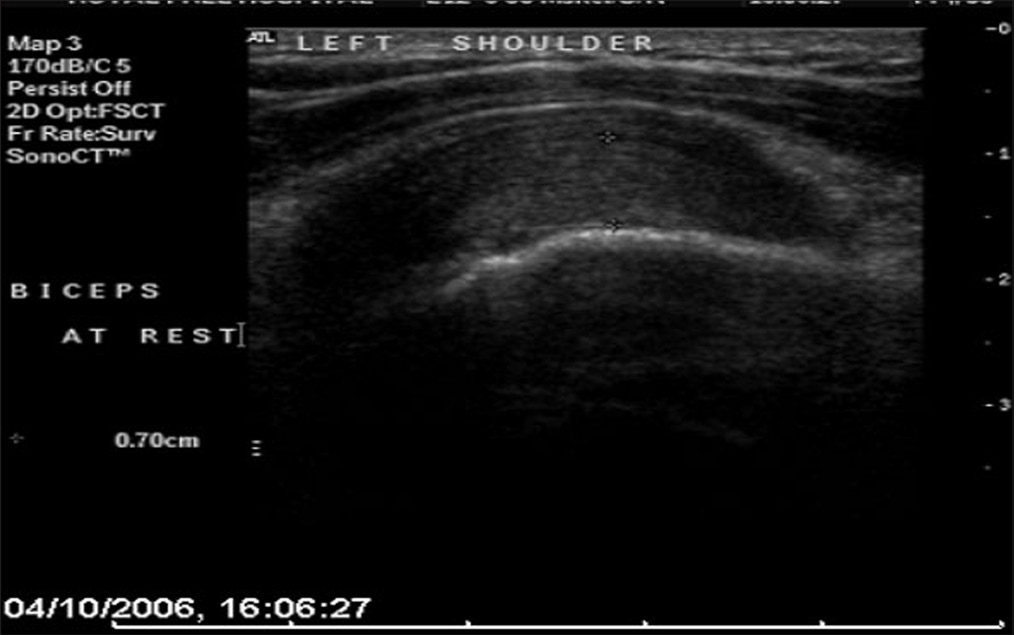
Ultrasound image of a longitudinal view of the LHB at rest

**Figure 4:**
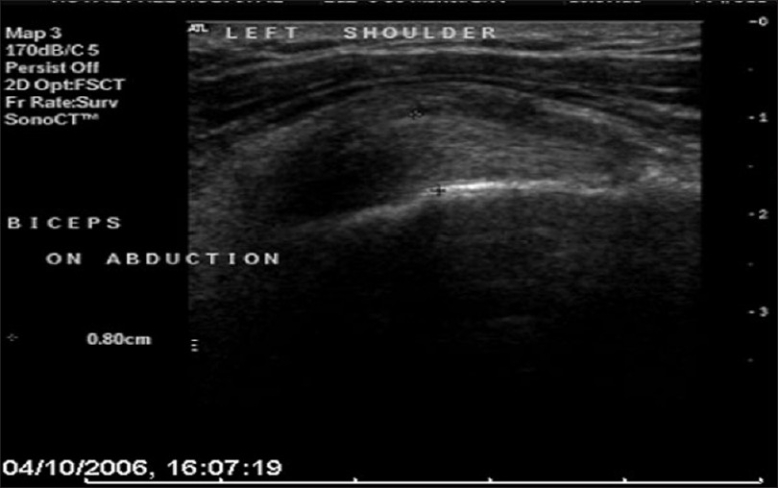
Ultrasound image of a longitudinal view of the LHB in abduction, demonstrating increased tendon diameter

**Figure 5:**
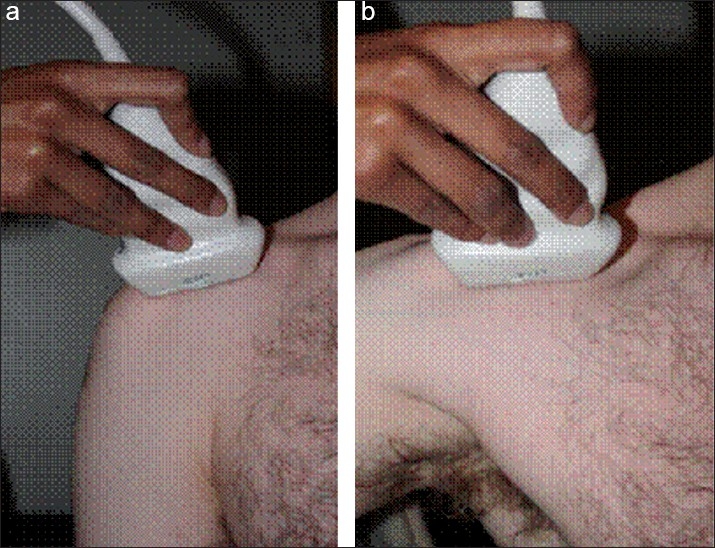
Clinical photographs demonstrating the ultrasound probe position, with the arm at the side (a), and on active abduction (b)

Eight patients were referred to a specialized musculoskeletal radiologist (BH) for dynamic U/S imaging. The first part of the examination was focused on assessing the integrity of the rotator cuff, the technique for which has been described previously. The biceps tendon was then assessed for the presence of tendinopathy and / or hypertrophy [Figure 3]. With the probe aligned in the long axis plane of the intra-articular portion of the biceps tendon, the arm was both actively and passively elevated [Figure 4]. A 10% increase in the diameter of the tendon, or tendon buckling was considered diagnostic of an hourglass deformity [Figure 5].

All the other cases (eight cases) had a standard U/S examination performed as described in earlier studies, and served as a control group. The choice of examination was governed by the availability of ultrasound appointments at the author's institution.

### Arthroscopic procedure

**Figure 6:**
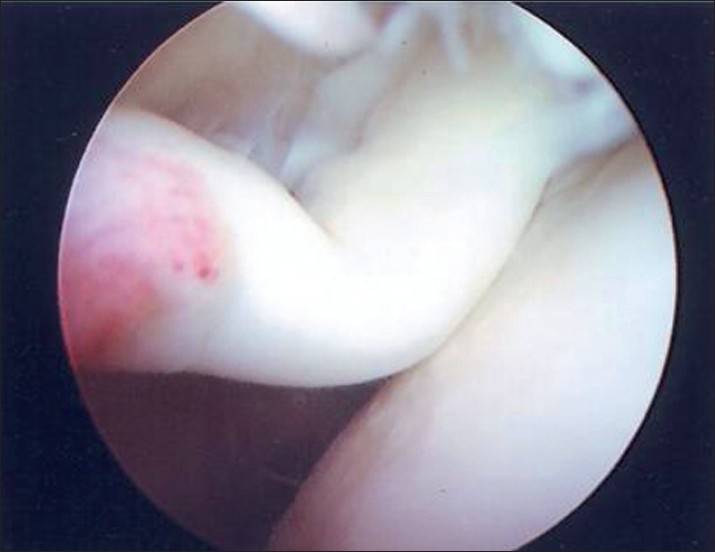
Arthroscopic photograph of LHB buckling during an hourglass test

Arthroscopic evaluation and treatment were performed by the senior surgeon (P.M.A). The beach chair position with a standard posterior viewing portal was used. The glenohumeral inspection started from the origin of the LHB. The aspect of the LHB was noted for: hypertrophy, fraying, and subluxation. Its intra-articular and intertubercular course was assessed with a probe. The intraoperative hourglass test was performed, elevating the arm in the plane of the scapula in neutral rotation with the elbow extended, to demonstrate buckling of the LHB [Figures 5 and 6]. After examination of the LHB, the articular side of the cuff tendons were inspected. Rotator cuff tears were classified by type (partial or full thickness) and size. The anterosuperior glenoid rim and labral structures were examined. The subacromial space was arthrocopically evaluated through the posterior portal.

The clinical preoperative evaluation, documented ultrasonographic findings, and intraoperative arthroscopic findings were recorded in a standardied fashion.

## RESULTS

The overall results are summarized in Table 1.

**Table 1:** LHB pathology. Dynamic versus standard ultrasonographic findings

	Standard ultrasound technique (n = 8)	Dynamic ultrasound technique (n = 8)
LHB abnormality	6	6
LHB hypertrophy	3	5
LHB entrapment / hourglass biceps	0	3

During arthroscopy, an hourglass biceps was identified with a hypertrophic intra-articular portion in all cases. Twelve patients had true-positive U/S findings of biceps tendon abnormality.

At surgery, all hourglass biceps except one were found to be associated with a rotator cuff tear. There one full thickness tear and three partial tears the subscapularis tendon.

Hypertrophy of the LHB was demonstrated in three out of eight patients by standard ultrasound and five out of eight patients with dynamic testing. One LHB dislocation was correctly identified. The hourglass biceps phenomenon was detected in three out of eight cases by dynamic U/S.

During the study period there was no case of a false positive dynamic ultrasound for the hourglass lesion.

All patients except one were treated by biceps tenotomy, after removal of the hypertrophic intra-articular portion of the tendon, ‘bipolar tenotomy’. Appropriate treatment of the concomittant lesions was simultaneously performed.

In one case, a 45-year-old woman with an isolated hourglass biceps, with an intact rotator cuff, was treated by arthroscopic tenodesis. She had previously undergone intramedullary nailing for a humeral fracture, which had been removed without relieving her shoulder symptoms. The findings were of a partial, probable iatrogenic tear of the LHB and a healed rotator cuff.

The number of cases involved in this preliminary study preclude meaningful statistical analysis, and therefore, the data has been left as absolute figures.

## DISCUSSION

Lesions of the tendon of the long head of the biceps tendon are a common cause of shoulder pain. Instability,[3] tendinitis, groove pathology,[15] pulley lesions,[16] and intrinsic degeneration are all well-recognied, and recently, intra-articular entrapment of the LHB has been described, causing pain and ‘locking’ of the shoulder, termed ‘the hourglass biceps’.[4] A number of symptoms and signs can suggest biceps tendon abnormality as the source of shoulder pain, but accurate clinical diagnosis remains difficult. In addition, LHB and rotator cuff lesions may frequently coexist and further complicate the clinical evaluation of these patients.

Imaging techniques may allow greater accuracy in diagnosing biceps pathology, allowing for appropriate treatment plans and better patient information before surgical intervention. The musculoskeletal has several advantages when compared with other imaging modalities, such as, computerised tomography, CT arthrography, magnetic resonnance (MR) imaging, and MR arthrography. It is non-invasive, is widely available, quick, less expensive, and allows dynamic assessment.[17]

Armstrong *et al*.[12] found a specificity of 100% and a sensitivity of 50% in determining LHB abnormalities by U/S in 71 patients, with prospective evaluation. They focused on subluxations and dislocations of LHB, but with a non-dynamic U/S evaluation. U/S detected none of the 23 partial-thickness tears of the LHB.

The capacity for dynamic assessment with U/S has been shown to improve the accuracy of biceps tendon location.[18,14] The dynamic effectiveness of U/S in determining a subluxation or a dislocation of the LHB has been published,[14] showing an accuracy rate of 86%.

Treatment of hourglass biceps, as described previously,[4] should either be by tenodesis[19,1] or a tenotomy,[20] with a resection of the hypertrophied portion, allowing the LHB to slide into the bicipital groove. If a simple tenotomy is performed, it may remain above the groove, and cause secondary impingement within the joint against the glenoid, or in the subacromial space with the acromion or coracoid process, if the tendon passes through an associated full-thickness cuff tear.

Our study confirms previous data showing the difficulty of accurately demonstrating biceps pathology by U/S. However, hypertrophy of the tendon did predict an hourglass biceps in eight out of the 16 cases. Dynamic testing identified an hourglass biceps in three out of eight cases, and increased the accuracy of detection tendon hypertrophy to five out of eight cases. Icreased awareness and experience, especially in conjunction with dynamic testing, improve the accurate preoperative diagnosis of LHB pathology and demonstrate the hourglass lesion preoperatively, although arthroscopy remains the gold standard.
